# Efficacy of virtual reality-based exposure therapy for post-traumatic stress disorder in military veterans: a systematic review and meta-analysis

**DOI:** 10.3389/fpsyt.2026.1857109

**Published:** 2026-08-05

**Authors:** Reema Ghazi Felemban, Rayan Rajab Alzahrani, Nawaf Fahad Alrefaei, Nawaf Muteb Alharbi, Abdulrahman Saleh Alghamdi, Saleh Alqadi

**Affiliations:** 1 College of Medicine, King Saud bin Abdulaziz University for Health Sciences, Jeddah, Saudi Arabia; 2 King Abdullah International Medical Research Center, Jeddah, Saudi Arabia; 3 Psychiatry Section, Department of Medicine, King Abdulaziz Medical City, Ministry of the National Guard-Health Affairs, Jeddah, Saudi Arabia

**Keywords:** clinical efficacy, combat trauma, military veterans, PTSD - Posttraumatic stress disorder, VRET-virtual reality exposure therapy

## Abstract

This systematic review and meta-analysis evaluated the effectiveness of Virtual Reality Exposure Therapy (VRET) for treating Post-Traumatic Stress Disorder (PTSD) in military veterans. A comprehensive search of electronic databases identified 9 randomized controlled trials involving 444 veterans that met inclusion criteria. The primary outcomes were changes in PTSD symptom severity measured by the Clinician-Administered PTSD Scale (CAPS) and PTSD Checklist (PCL). The meta-analysis demonstrated significant reductions in PTSD symptom severity in pre–post comparisons following VRET. In contrast, comparisons between VRET and control conditions showed smaller and more variable effects, highlighting the importance of cautious interpretation when assessing comparative efficacy. The pooled mean difference in CAPS scores was 33.73 (95% CI: 7.80 to 59.65) for pre-post comparisons, indicating a marked reduction in symptom severity following treatment. For PCL scores, the pooled mean difference was 20.96 (95% CI: 13.05 to 28.86). When compared to controlled conditions, VRET was associated with modest effects, with a pooled mean difference of 4.86 (95% CI: -8.34 to 18.07) for CAPS scores and 1.93 (95% CI: - 0.02 to 3.89) for PCL scores. Overall, combining pre-post and controlled comparisons yielded statistically significant effects favoring VRET, with mean differences of 23.26 (95% CI: 8.00 to 38.52) for CAPS and 13.66 (95% CI: 5.00 to 22.31) for PCL scores. However, high heterogeneity was observed across studies, warranting cautious interpretation. Risk of bias assessment indicated low to moderate risk of bias across the included studies. Visual inspection of funnel plots suggested no strong asymmetry; however, interpretation is limited due to the small number of studies included. These findings suggest that VRET may be an effective intervention for reducing PTSD symptoms in military veterans, particularly in within-group analyses, although comparative effects relative to control conditions were more modest. The immersive features of VRET may offer an alternative means of delivering exposure-based interventions, although patient acceptability and clinical utility likely vary across individuals and settings. VRET shows promise as an alternative or adjunct to conventional treatments, potentially improving engagement and reducing dropout rates. However, small sample sizes in some studies and heterogeneity of results limit definitive conclusions. Further large-scale trials are needed to establish VRET’s comparative efficacy and long-term benefits in military populations.**Aim**: To systematically review and meta-analyze the efficacy of virtual reality-based exposure therapy (VR-ET) for treating post-traumatic stress disorder (PTSD) in military veterans.**Objectives:** To evaluate the efficacy of VR-based exposure therapy in reducing PTSD symptoms and achieving remission among military veterans. 2. To compare the efficacy of different VR protocols, such as: Immersive vs. non-immersive systems, Combat-related vs. non-combat trauma scenarios 3. To explore heterogeneity in treatment outcomes based on: Veteran-specific factors (e.g., age, service duration, comorbidities)Study characteristics (e.g., sample size, design, control group) 4. To identify barriers to implementation of VR-based exposure therapy in veteran healthcare systems, including: Cost and resource availability, Technological literacy among patients and providers, Logistical and infrastructural limitations

**Systematic Review Registration:**

https://www.crd.york.ac.uk/PROSPERO/view/CRD420251207784, identifier CRD420251207784.

## Introduction

Post-Traumatic Stress Disorder (PTSD) has emerged as prevalent and devastating psychiatric disorders affecting military veterans from all over the globe (1). PTSD is characterized by hyperarousal, avoidance behaviors, intrusive memories, and emotional numbing due to frequent exposure to life threatening events that significantly impair the quality of life and restrict successful acceptance of civilian life. Approximately, 11-20% of veterans served in operations such as Iraqi Freedom and Enduring Freedom suffers from severe PTSD as reported by U.S. Department of Veterans Affairs (2). Many active-duty militants don’t adopt these treatments or sometimes discontinue them due to poor engagement, stigma or psychological burden linked with traumatic memories despite of continuous availability of psychological treatments, such as prolonged exposure therapy (PE), cognitive behavioral therapy (CBT), and other pharmacotherapy (3).

With technological advancements, virtual reality-based exposure therapy, or VRET, has emerged as digital health innovation from last two decades. This therapeutic approach has gained attention due to drawbacks of conventional PTSD treatments for military veterans (4, 5). VRET applies realistic and computer-generated simulations to reconstruct the traumatic situation in a safe and regulated context for PTSD treatment (3). By providing multimodal stimuli, such as tactile, visual, and auditory feedback, VRET allows for graded, personalized exposure to signals associated with trauma under the supervision of a therapist (6). This immersive exposure aims to reduce fear responses and avoidance behaviors by utilizing emotional processing and habituation mechanisms (7). Importantly, tech-savvy veterans who might normally be reluctant to engage in traditional talk therapy could find VRET to be a more engaging and dynamic alternative (8, 9).

Various studies have reported the effectiveness of VRET in different therapeutic settings for treatment of sexual assault survivors, veterans and phobia-stricken citizens who served in war (10, 11). While some clinical trials report considerable gains in treatment adherence and a significant reduction in the severity of PTSD symptoms, others show minimal effects or highlight logistical and technological limitations (12). Veterans may respond differently to VRET depending on their degree of technological comfort, views on treatment realism, and prior experience with combat simulators. Furthermore, there is still substantial variation in the research on the effectiveness of VRET for PTSD in military veterans. While previous meta-analyses have pooled mixed civilian and military cohorts, which often masks unique clinical complexities and high treatment resistance inherent to combat-related trauma (4, 5), a dedicated synthesis focusing exclusively on the veteran subpopulation remains essential. Moreover, rapid advancements in virtual reality architectures over recent years warrant an updated longitudinal evaluation of the literature. Thus, the goal of this systematic review and meta-analysis is to thoroughly assess VRET’s effectiveness in treating PTSD in veterans of the armed forces.

## Methods

### Search design

This meta-analysis was performed by following “Reporting Items for Systematic Review and Meta-Analysis (PRISMA)” guidelines (13) to fulfill research aims. There is no need for an additional ethical review due to the involvement of previously published retrospective and prospective cohort studies.

### PICO framework

This study used the Population Intervention Control Outcome Study (PICOS) framework to guide the search.

P (Population): Military veterans diagnosed with Post-Traumatic Stress Disorder (PTSD), as defined by standard diagnostic criteria (e.g., DSM-IV, DSM-5, ICD-10).

I (Intervention): Virtual Reality-Based Exposure Therapy (VRET), including both fully immersive and semi-immersive formats, used as a therapeutic intervention for PTSD.

C (Comparison): Standard care (e.g., cognitive behavioral therapy, prolonged exposure therapy), waitlist controls, or no treatment.

O (Outcomes): Change in PTSD symptom severity (measured by validated PTSD scales such as CAPS, PCL, or IES-R).

### Search strategy

Three Electronic databases, PubMed, APA PsychNet, and Cochrane Library, were searched from inception to April 2025. The MeSH keywords used for search of research articles from PubMed were (“Virtual Reality”[Mesh] OR “virtual reality” OR “virtual reality exposure therapy” OR “VRET” OR “VR therapy” OR “immersive therapy” OR “VR-based therapy”) AND (“Stress Disorders, Post-Traumatic”[Mesh] OR “post-traumatic stress disorder” OR “PTSD” OR “combat stress” OR “war trauma”) AND (“Veterans”[Mesh] OR “military personnel” OR “military veteran*” OR “combat veteran*” OR “ex-military” OR “armed forces”) AND (“Treatment Outcome”[Mesh] OR “efficacy” OR “clinical effectiveness” OR “therapy outcome” OR “treatment response” OR “patient satisfaction” OR “adherence” OR “ OR “retention”). Similar search strategy was used for other databases. The search was restricted to English language. These three databases (PubMed, PsycNet, Cochrane) were used selectively because they represent the largest number of relevant articles regarding PTSD and digital interventions from both a medical and psychological perspective. In addition to using these databases, manual forward and backward citation searches of the references included in each eligible study and review article were conducted to help reduce any possible publication bias.

### Eligibility criteria

The eligibility criteria were used to select and screen research articles after searching for research articles from electronic databases.

#### Inclusion criteria

1. Studies analyzed the military veteran population (>18 years) diagnosed with PTSD by DSM-IV, DSM-5, ICD-10
2. Studies evaluated the effects of Virtual Reality-Based Exposure Therapy (VRET) in comparison to control
3. Studies tracking patient outcomes related to the change in severity of PTSD symptoms
4. Primary research studies such as randomized controlled trials (RCTs)
5. Studies with full text available and published in English language.

#### Exclusion criteria

Those studies were excluded:

1. Studies analyzing the public or students or other civilian adults with PTSD
2. Studies analyzing patient receiving other traditional behavioral or cognitive therapies
3. Studies based on systematic review, meta-analysis, comprehensive reviews, narrative reviews, case-control studies, and editorials
4. Studies published in other languages rather than English and non-full text papers.

### Data extraction

Two blinded reviewers independently screened studies for inclusion, and any disagreements were resolved through discussion or consultation with a third reviewer to ensure consistency and reduce selection bias. The studies obtained by the database search were entered into the EndNote library. Duplicates were excluded in the next step. While a formal inter-rater agreement coefficient (Cohen’s Kappa, k) was not computationally recorded during the screening tracking phase, high screening alignment was maintained through strict adherence to the predefined eligibility criteria. The eligibility criteria were applied by reviewers in a blinded manner to all individual studies. Discrepancies were sorted by mutual agreement. Data related to demographic information, such as authors, year, country, study population, study design, study follow up and primary outcomes, such as severity in PTSD symptoms were extracted (**Table 1**). Discrepancies were resolved by consulting with a third reviewer.

**Table 1:** Characteristics of included studies.

Author, year	Country	Study population	Study design	Follow up	Type of VR	Change in PTSD symptom severity (CAPS)	WHO- five well- being index	Hamilton depression scale	PTSD symptoms (PCL)
Folke et al., 2023 (14)	Afghanistan	9 Danish veterans with PTSD	Mixed Method pilot study	3 months	10 sessions of BraveMind virtual reality exposure therapy (VRET)	Pre: 34.33 (8.44) Post: 22.50 (7.94)	Pre: 12.57 (9.64) Post: 21.33 (4.62)		Pre: 62.86 (10.48) Post: 51.75 (15.77)
Beidel et al., 2019 (15)	Afghanistan and Iraq	92 Afghan and Iraq veterans (36.7 years) T1: 49 T2: 43	Randomized controlled trial	6 months	Virtual reality exposure therapy	Pre: 85.5 (17.7) Post: 41.8 (24.5)		Pre: 25.3 (9.6) Post: 13.2 (7.8	Pre: 63.8 (11.6) Post: 38.5 (13.9)
Reger et al., 2011 (16)	Iraq or Afghanistan	24 active duty soldiers with PTSD T1: 12 T2: 12	Pilot study	1 month	virtual reality exposure therapy (VRE) vs control exposure therapy (CET)				Pre: 60.92 (11.03) Post: 47.08 (12.70)
McLay et al., 2011 (17)	Iraq and Afghanistan	20 active duty soldiers (28.8 years)	Randomized controlled trial	10 weeks	VR-graded exposure therapy	Pre: 68 Post: 7			
McLay et al., 2017 (18)	Iraq and Afghanistan	85 active duty soldiers with PTSD (32.5 years) T1: 42 T2: 43	Randomized controlled trial	3 months	Virtual reality exposure therapy (VRET) vs control exposure therapy (CET)	T1: 11.0 (24.4) T2: 18.0 (33.7)			
Ready et al., 2010 (19)	Vietnam	11 veterans with PTSD (57 years) T1: 6 T2: 5	Randomized controlled trial	6 months	Virtual reality exposure therapy (VRET) vs present- centered therapy (PCT)	T1: 25.0 (28.1) T2: 13.0 (11.3)			
Miyahira et al., 2012 (20)	Iraq and Afghanistan	22 active duty service members with PTSD T1: 10 T2: 12	Randomized controlled trial	5 weeks	virtual reality exposure therapy (VRE) vs minimal attention	T1: 14.9 (5.29) T2: 4.1 (6.90)			T1: 6.17 (3.9) T2: 5.25 (2.02)
Loucks et al., 2019 (21)	Iraq and Afghanistan	15 veterans with PTSD	Randomized controlled	3 months	BraveMind VRE therapy	Pre: 41.53 (10.08)			Pre: 48.35 (13.48)
			trial			Post: 27.89 (14.63)			Post: 35.00 (18.37)
Reger et al., 2016 (22)	Iraq and Afghanistan	162 active duty soldiers T1: 18 T2: 24	Randomized controlled trial	6 months	10 sessions of virtual reality exposure therapy (VRE) vs Prolonged exposure	Pre: 15.46 [4.18, 26.74] Post: 14.43 [2.20, 26.65]			T1: 2.86 [- 2.58, 8.29] T2: -0.06 [- 6.02, 5.90]

PTSD: post-traumatic stress disorder, CAPS: Clinician-Administered PTSD Scale, PCL: Post-traumatic Stress Disorder Checklist.

### Risk of bias assessment

The risk bias of the included RCTs was evaluated using the Cochrane risk of bias method. Six domains—allocation concealment, participant blinding, selection bias, outcome assessment blinding, selective reporting, and additional bias—were used to assess the risk bias of the included research. Each included study’s score or level was divided into three categories: low risk, unclear, and high risk (23).

### Statistical analysis

Statistical analyses were performed using Review Manager Software (Cochrane Collaboration, version 5.4.0). For studies with possible heterogeneity, pooled data analysis was carried out using random effects models. P <0.05 was deemed statistically significant, which was the threshold for statistical significance. The I2 statistic was used to assess heterogeneity; considerable heterogeneity was indicated by I2 values more than 50% (24).

## Results

### Study selection

**Figure 1:**
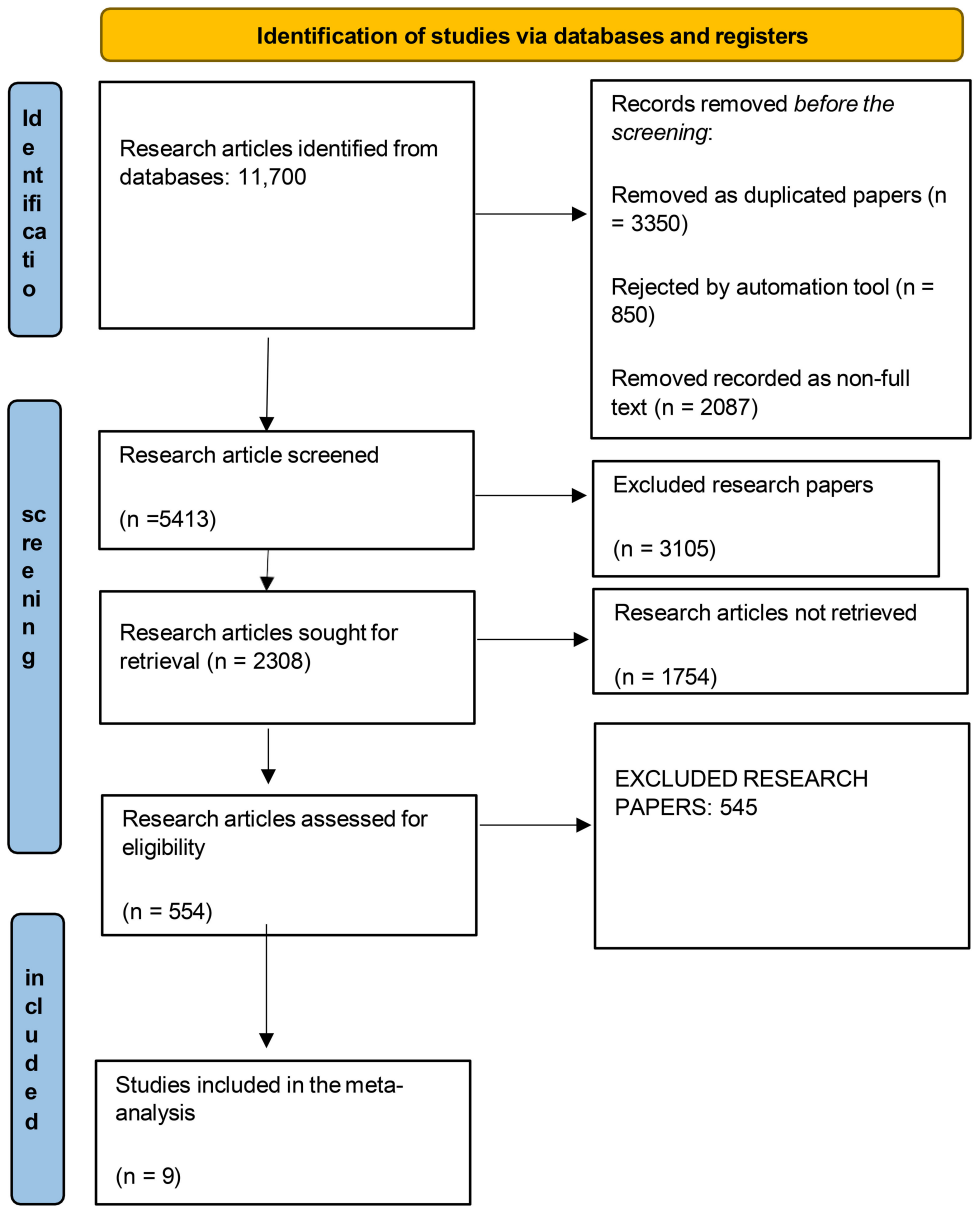
PRISMA flow chart for screening and selection of included studies.

The selection and screening of research articles related to the study aim was performed by following the PRISMA guidelines in this meta-analysis. Total of 11,700 records were generated after database searches and 5413 remained after removal of duplicates as well as non-full text. Total 5413 research articles were initially screened, and 2308 papers were sought for retrieval. Only 554 papers were assessed for eligibility criteria, and the final number of research articles was 9. In total, this meta-analysis is based on 9 RCTs studies, as mentioned in **Figure 1**.

### Risk bias assessment

**Figure 2:**
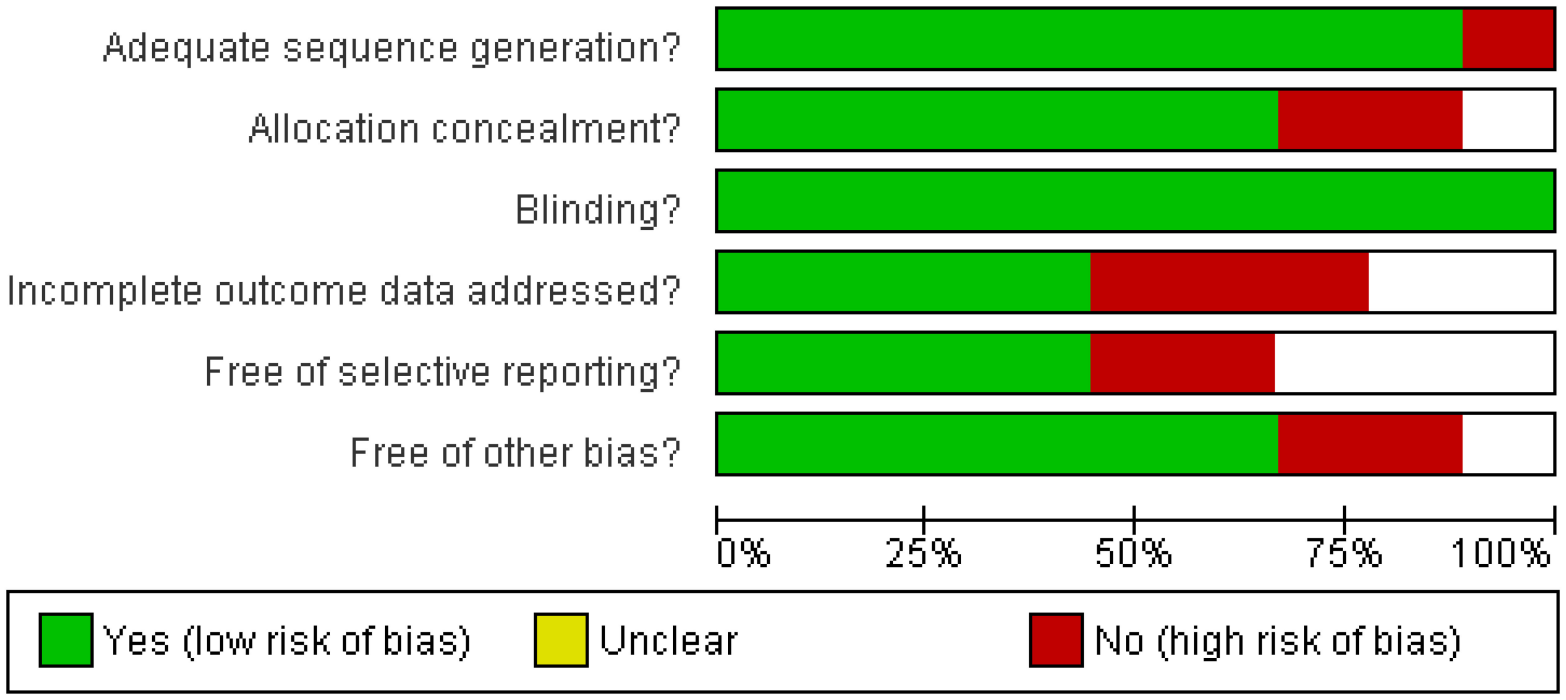
Risk of bias graph of included studies.

**Figure 3:**
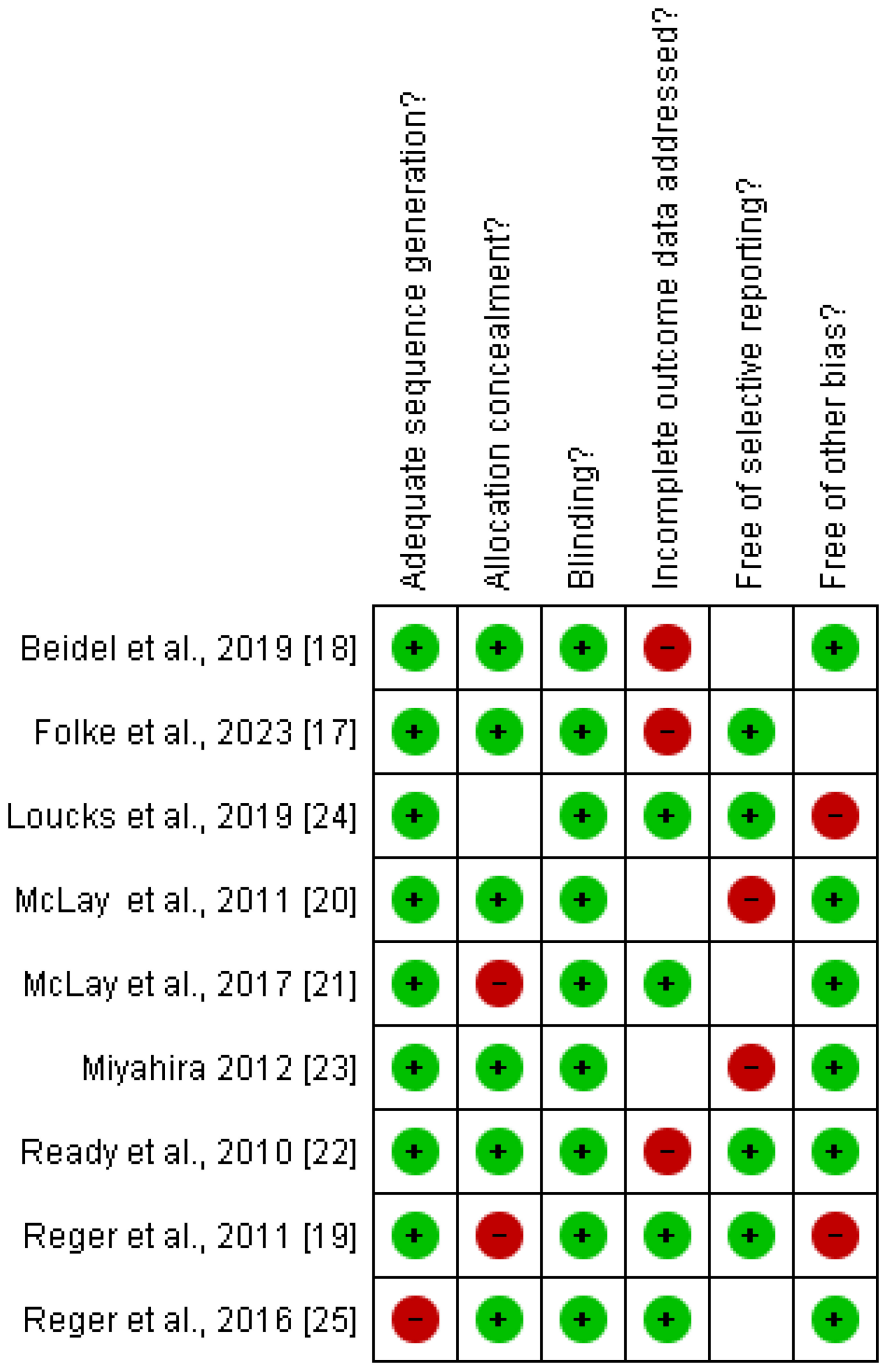
Risk of bias summary of included studies.

The Cochrane tool was used for risk bias assessment of all included RCTs. The purpose of using this Cochrane tool was the evaluations of whole study’s risk bias rather than methodological quality of each study. All included studies were low to moderate risk, and no study was high risk, as mentioned in **Figures 2**, **3**.

## Primary outcomes

### Change in severity of PTSD symptoms (CAPS)

**Figure 4:**
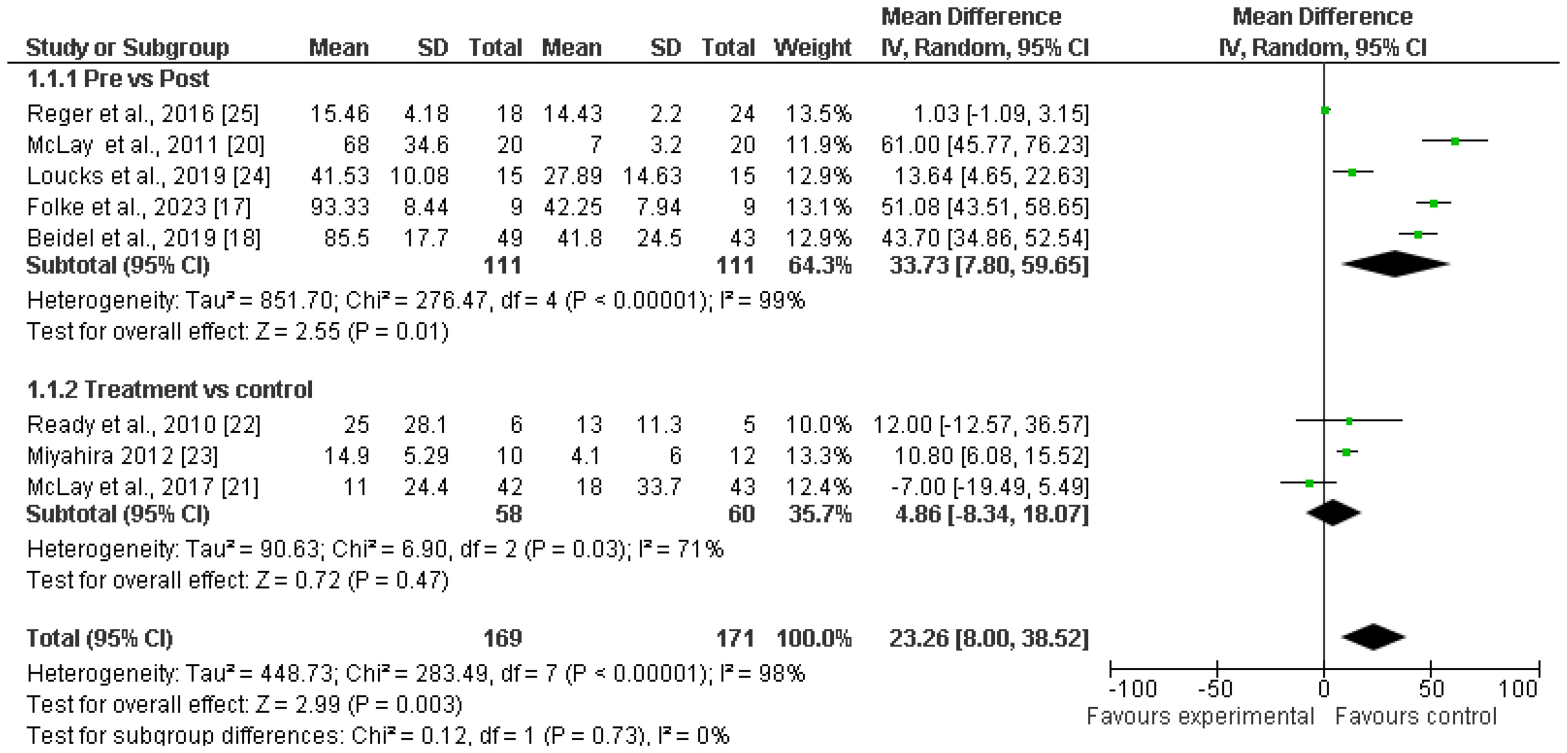
Forest plot of mean difference of CAPS scores among treatment (post) and placebo (pre) groups.

**Figure 5:**
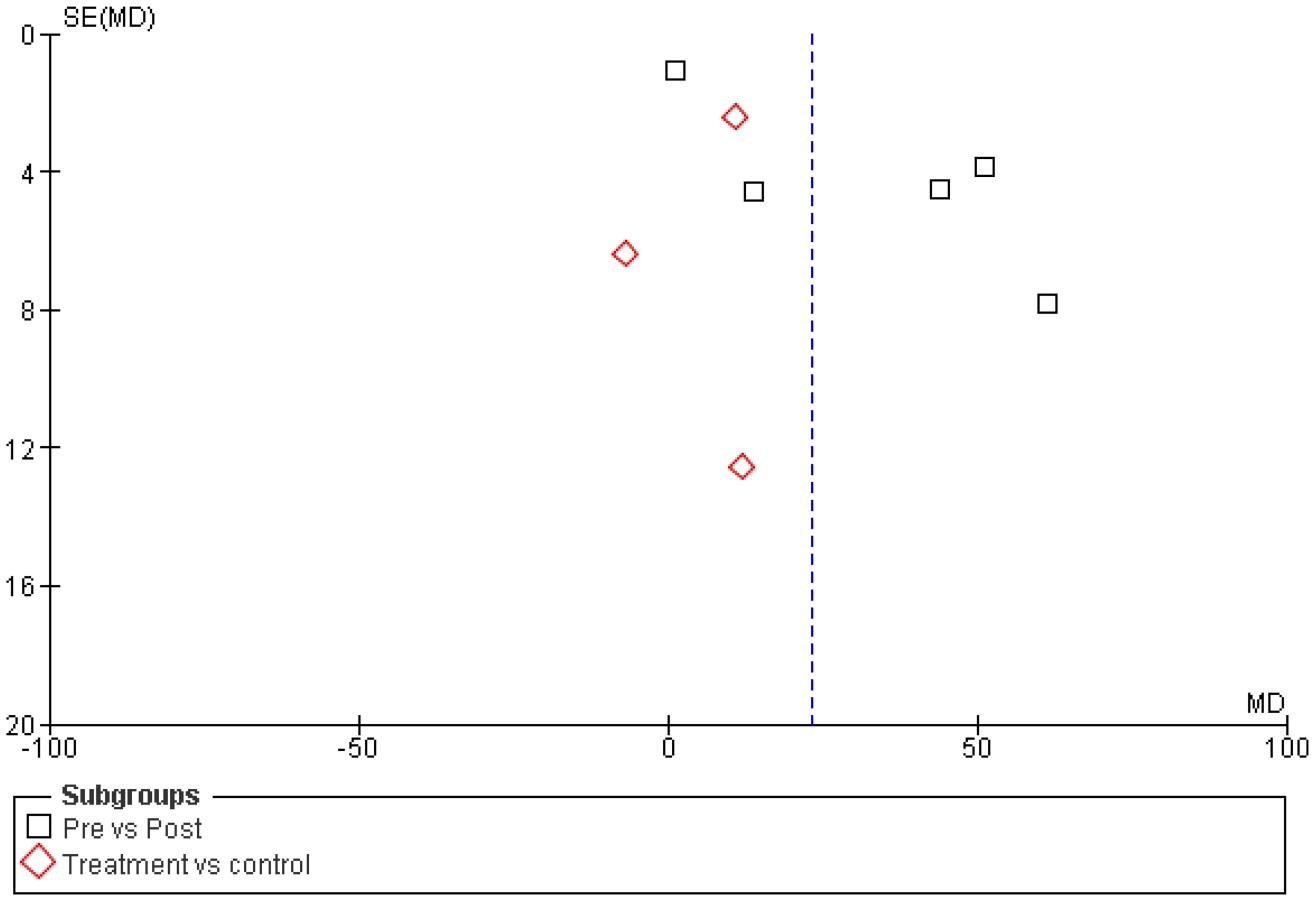
Funnel plot of mean difference of CAPS score among treatment (post) and control (pre) groups.

The forest plot (**Figure 4**) illustrates the effectiveness of Virtual Reality Exposure Therapy (VRET) for PTSD in military veterans. In the Pre vs Post subgroup (five studies), VRET led to a significant reduction in PTSD symptoms, with a pooled mean difference of 33.73 (95% CI: 7.80 to 59.65), indicating strong improvement after treatment. High heterogeneity was present among the included studies (I² = 99%, Tau² = 851.70), reflecting major variability in effect sizes across trials. Such high heterogeneity is not surprising for psychological and technology-based interventions such as VRET, given the variability in session structure, realism of virtual environments, and control conditions that exist across studies. For the sensitivity analysis, the study with the highest treatment effect size was excluded (Loucks et al., 2019). The removal led to a reduction of I² to 82% and resulted in a pooled estimate in the same direction at statistical significance. These findings suggest that, while there is variation across studies, the overall effect of VRET was associated with reductions in PTSD symptom severity in pre–post analyses. Nevertheless, the results should be interpreted with caution, and future trials should standardize intervention parameters to reduce heterogeneity. In contrast, the Treatment vs Control subgroup (I² = 71%, Tau² = 90.63; three studies) showed a non- significant effect with a pooled mean difference of 4.86 (95% CI: -8.34 to 18.07) and Z = 0.72 (P= 0.47), indicating no clear benefit of VRET over other interventions or controls. Overall, combining both subgroups resulted in a statistically significant total effect (mean difference: 23.26; 95% CI: 8.00 to 38.52), suggesting a potential beneficial effect of VRET on PTSD symptom severity. However, the high heterogeneity (I² = 98%, Tau² = 448.73) and subgroup differences (P = 0.73) warrant cautious interpretation and further exploration. **Figure 5** presents the funnel plot for CAPS scores.

### Change in severity of PTSD symptoms (PCL)

**Figure 6:**
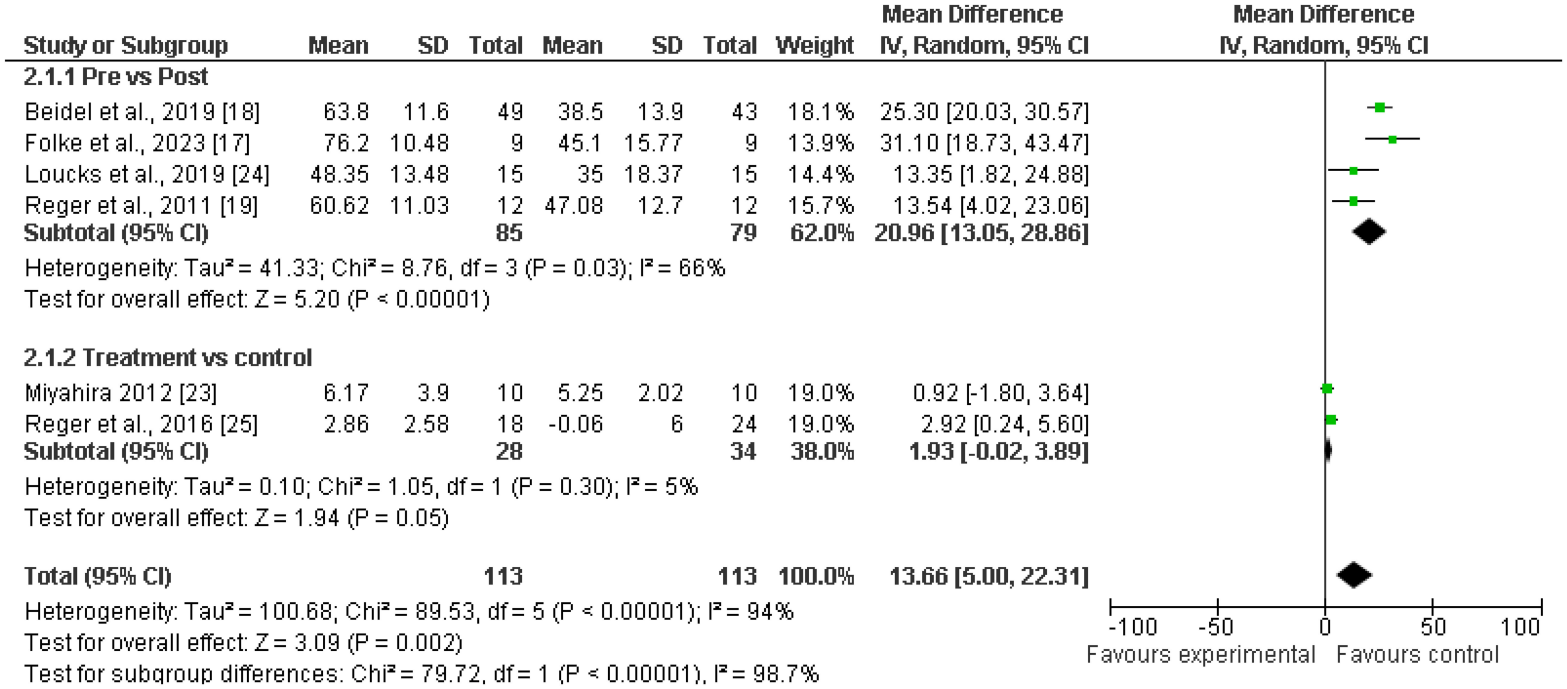
Forest plot of mean difference of PCL score among treatment (post) and control (pre) groups.

**Figure 7:**
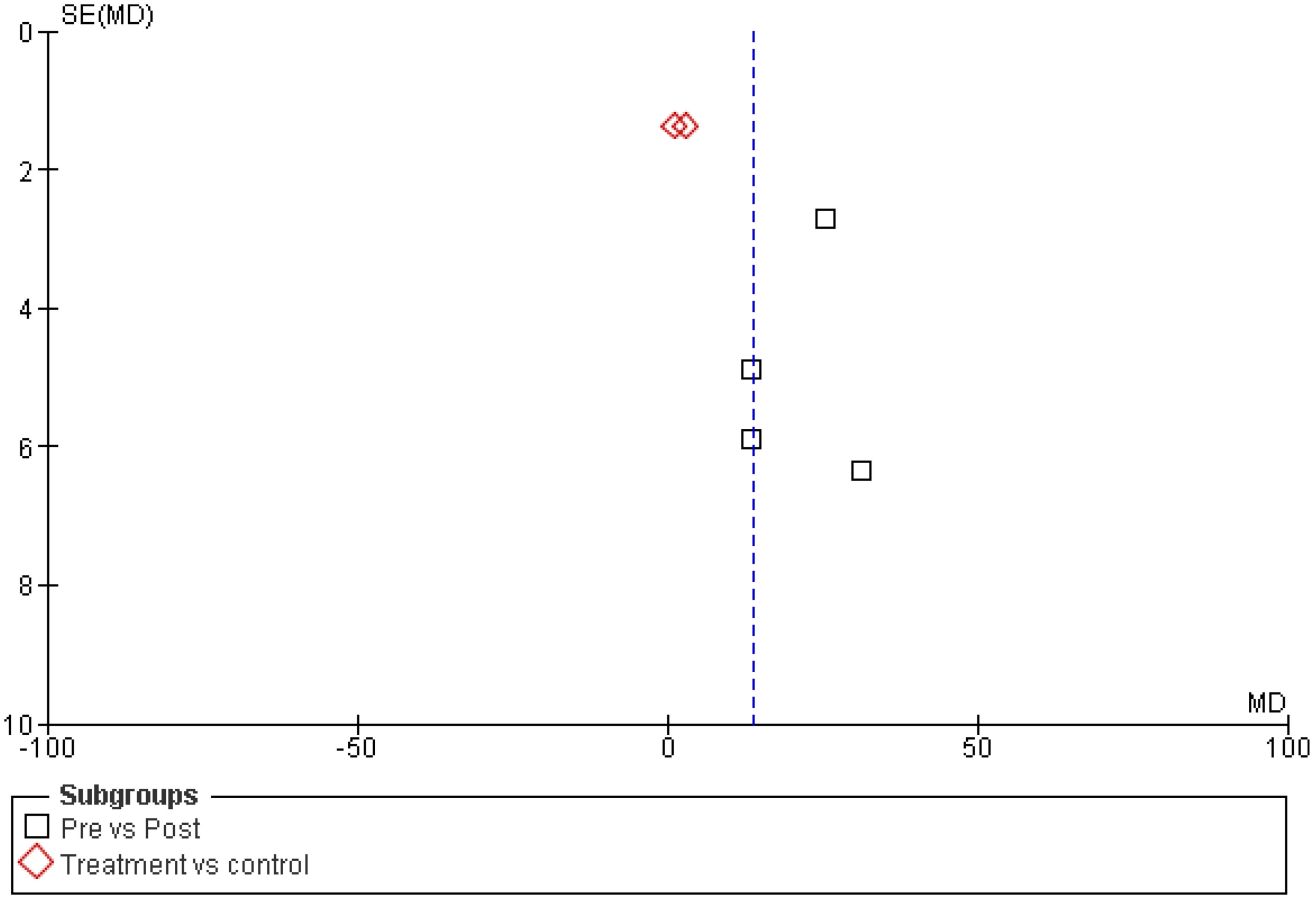
Funnel plot of mean difference of PCL score among treatment (post) and control (pre) groups.

This forest plot (**Figure 6**) presents findings from studies assessing the effects of Virtual Reality Exposure Therapy (VRET) for PTSD in military veterans, comparing pre- vs post-treatment and treatment vs control outcomes. In the Pre vs Post subgroup (4 studies), VRET significantly improved PTSD symptoms with a pooled mean difference of 20.96 (95% CI: 13.05 to 28.86), indicating a notable reduction in symptom severity following therapy. The test for overall effect was significant (Z = 5.20, P < 0.00001), although moderate heterogeneity was present (I² = 66%, Tau² = 41.33), suggesting some variability among study results. In the Treatment vs Control subgroup (2 studies), the pooled effect showed a smaller and borderline significant mean difference of 1.93 (95% CI: -0.02 to 3.89), with Z = 1.94 (P = 0.05), indicating a slight edge for VRET over control, though not definitively conclusive. Heterogeneity was low (I² = 5%, Tau² = 0.10). Overall, combining both subgroups resulted in a statistically significant pooled mean difference of 13.66 (95% CI: 5.00 to 22.31) with high heterogeneity (I² = 94%, Tau² = 100.68), indicating overall symptom improvement associated with VRET, while highlighting considerable variability across studies. **Figure 7** presents the funnel plot for PCL scores.

## Discussion

This study aimed to evaluate the effects of Virtual Reality-Based Exposure Therapy for Post- Traumatic Stress Disorder in Military Veterans by adopting meta-analysis research approach. Through 9 RCTs and 444 military veterans, the findings revealed that significant change in severity of PTSD symptoms in form of CAPS and PCL scores have been reported after virtual Reality Exposure Therapy (VRET) as compared to control. The pooled analysis showed that VRET improved the CAPS (23.26; 95% CI: 8.00 to 38.52), and PCL (13.66; 95% CI: 5.00 to 22.31) scores among military veterans. Furthermore, the symmetrical distribution of studies on funnel plot suggested low to moderate publication bias among included studies. The methodological quality and risk of bias of the included randomized controlled trials (RCTs) are summarized in **Figure 3**. While a substantial portion of the domains demonstrated a low risk of bias, high or unclear risk of bias was observed in specific trials, particularly regarding allocation concealment and selective reporting. These variations highlight methodological variations across the literature but do not compromise the overall trends observed in the clinical outcomes.

The meta-analysis’s findings suggest symptoms improvement following VRET rather than definitive superiority over standard treatments. VRET can result in considerable reductions in the severity of PTSD, as demonstrated by the notable improvements observed in the pre- and post-treatment comparisons. These findings are in line with past research that showed VRET to be a promising therapeutic technique for traumatized people, especially in military populations (25–27). Compared to traditional imaginal exposure, VRET’s immersive nature enables more regulated and adaptable exposure to trauma-related stimuli, which may boost emotional engagement and speed up desensitization.

Clinical research supports the use of VRET as a substitute or supplement to conventional exposure-based treatments, especially for veterans who are reluctant to participate in imaginal exposure or who need more individualized, engaging interventions. In military settings, where stigma and treatment dropout rates are still high, its potential to increase patient participation and decrease avoidance behaviors is particularly pertinent. The results also imply that VRET could be a useful instrument in stepped-care approaches, providing a compromise between challenges of psychotherapy and medication.

However, several limitations must be acknowledged. The small sample sizes in many included studies limit statistical power and may inflate effect estimates. Furthermore, blinding is difficult to achieve in psychotherapy trials involving immersive technologies, potentially introducing performance or detection bias. The high heterogeneity observed in the pooled analysis suggests caution in interpreting the overall effect size, and more robust subgroup analyses are warranted to explore sources of variability. Considerable statistical heterogeneity was observed in the pooled analyses for both primary outcomes (I²> 90\% for CAPS and PCL analyses). While investigating potential moderators—such as immersion level architectures or specific session numbers—via a formal subgroup stratification or meta-regression would be methodologically ideal to explore this variability, it was restricted in the present study. Because the comprehensive systematic selection yielded a final sample size of 9 eligible randomized trials, performing a formal quantitative moderator analysis or multi-layered meta-regression would lack the baseline statistical power required to produce reliable estimates and could risk overfitting the data. Consequently, a random-effects framework was appropriately utilized to account for parameter variance, and a localized sensitivity analysis via systematic outlier exclusion was prioritized as the most statistically sound approach to examine effect stability. Additionally, few studies assessed long-term outcomes, leaving questions about the durability of treatment effects. Despite these limitations, this meta-analysis contributes valuable insights into the therapeutic potential of VRET and underscores the importance of continued research, particularly into optimizing intervention design, delivery, and integration into veteran healthcare systems.

### Practical implementation and clinical challenges

The integration of evidence-based practice from Virtual Reality Exposure Therapy (VRET) into standard psychiatric care settings represents several separate translational barriers. At a structural level, the cost of implementing high-fidelity head-mounted display devices (HMDs), in addition to the continuous costs associated with device maintenance, will require significant budgetary allocations by the Veterans Administration’s healthcare system (15, 18).

Beyond the costs associated with hardware and infrastructure, data indicate that effective delivery of VRET depends on access to well-trained mental-health professionals. In most trials, VR exposure was provided by professionals who either held some experience in working with trauma cases or received specialized training in trauma-focused interventions like Prolonged exposure. Additionally, VR-specific training was required to cover simulator operation, cybersickness monitoring, and in-session adjustment of parameters during the processing of traumatic memories (15, 17, 18, 22).

Moreover, given that veteran populations typically exhibit complex clinical profiles – which are often complicated by co-existing conditions including severe depression and/or substance abuse disorders – the potential impact that these factors may have on the patient’s course of treatment, their symptomatic responses, and ultimately their ability to retain and maintain long term improvement (16, 22) makes it evident that, although VRET has been demonstrated to be effective in structured research settings, its application in real world clinical environments will depend upon the creation of comprehensive clinical protocols and establishment of solid institutional support.

## Conclusion

The findings of this study support the potential efficacy of Virtual Reality Exposure Therapy (VRET) in reducing PTSD symptoms among military veterans, particularly when comparing pre- and post- treatment outcomes. While comparisons with control groups showed modest effects, the overall findings indicate that VRET may represent a useful adjunct to established exposure-based therapies for some individuals, particularly in contexts where imaginal exposure is difficult to engage with. However, high heterogeneity and limited standardized protocols highlight the need for further high-quality, large-scale studies. Future research should aim to refine intervention strategies, assess long-term effectiveness, and explore patient-centered outcomes to ensure optimal integration of VRET into clinical practice for veterans with PTSD.

## Data Availability

Publicly available datasets were analyzed in this study. This data can be found here: All datasets generated and analyzed during the current study are derived from published articles and are included in this article and its supplementary files.
